# Modulation of immune cells and metabolic reprogramming in efferocytosis

**DOI:** 10.1038/s41419-026-08431-8

**Published:** 2026-02-25

**Authors:** Karen Cristina Oliveira, Caroline Maria Marcos, Letícia de Aquino Penteado, Naiara Naiana Dejani, Pedro M. Moraes-Vieira, Alexandra I. Medeiros

**Affiliations:** 1 Department of Biological Sciences, School of Pharmaceutical Sciences, São Paulo State University (UNESP), Araraquara, São Paulo Brazil; 2 Laboratory of Biology, Control and Surveillance of Insect Vectors, Oswaldo Cruz Institute, Fiocruz, Rio de Janeiro Brazil; 3 Department of Biomedical Sciences, Federal University of Paraíba (UFPB), João Pessoa, Brazil; 4 Department of Genetics, Microbiology and Immunology, and Experimental Medicine Research Cluster (EMRC), State University of Campinas (UNICAMP), Institute of Biology, Campinas, Brazil

**Keywords:** Cell death and immune response, Infection

## Abstract

Under physiological conditions, cell apoptosis is a silent death process during tissue renewal and remodeling. The phagocytosis of apoptotic cells, known as efferocytosis, is a key process performed primarily by macrophages and dendritic cells, as well as by non-professional phagocytes, such as epithelial cells and fibroblasts. This process, which involves the removal of apoptotic cells, is not just a routine task. It plays a significant role in producing anti-inflammatory mediators that are instrumental in maintaining tissue homeostasis. However, during infection, pathogens can induce different patterns of cell death, including apoptosis. Efferocytosis of infected apoptotic cells is a crucial part of the host defense mechanism. It aids in bacterial clearance, activates the effector functions of phagocytes, and directs the activation of CD4+T lymphocytes. The different stages of the efferocytosis process are not just a sequence of events, but a complex interplay that can interfere with the microenvironment by releasing soluble mediators (“find-me signals”) as a rich source of nutrients for phagocytes during the digestion process (“digest-me”), such as amino acids, nucleotides, lipids, and carbohydrates. In recent years, several studies have contributed to unraveling the impact of the different stages of the efferocytosis process on regulating metabolic pathways that support the continuous phagocytosis of apoptotic cells, the activation profile, and the effector functions of phagocytes. In this review, we discuss the impact of efferocytosis on immune cells during homeostasis and infectious diseases, and in the metabolic reprogramming on phagocyte activation. We also explore the role of efferocytosis during the clearance of apoptotic cells in different pathologies.

## Facts

1. Efferocytosis is crucial for maintaining homeostasis, promoting tissue repair, and regulating the immune response.
2. Phagocyte recognition of apoptotic cells promotes an anti-inflammatory microenvironment.
3. Pathogen patterns inside apoptotic cells dictate the polarization of Th cells.
4. Efferocytosis of apoptotic cells activates metabolic pathways that drive the differentiation of tolerogenic antigen-presenting cells.

## Open Questions

1. Are there metabolic differences between professional and non-professional efferocytes during apoptotic cell clearance?
2. How does engulfing different types of dying cells (necrotic, apoptotic, ferroptosis) impact phagocytes’ metabolism?
3. How could recognition of different pathogen within of apoptotic cells impact the activation of metabolic pathways in the phagocytes?

## Introduction

Cell death is an essential biological process in both physiological and pathological contexts. It can be classified into programmed cell death (apoptosis, necroptosis, pyroptosis) and non-programmed death (conventional necrosis, ferroptosis). Those types of cell death differ in their morphological, biochemical, and molecular characteristics, as well as in their cellular outcomes. Apoptosis, a form of programmed cell death, occurs through extrinsic and intrinsic pathways. Intrinsic apoptosis is caused by DNA damage or mitochondrial damage that allows the release of cytochrome c as well as second mitochondria-derived activator of caspase (SMAC). On the other hand, extrinsic apoptosis is mediated by different molecules and ligands such as TNFR1-TNF, FAS-FasL, and TRAIL-DR4/DR5. Both pathways culminate in the activation of caspases 3 and 7, which promote DNA fragmentation and loss of viability, respectively, leading to apoptosis. Membrane integrity is initially preserved, with regulated phosphatidylserine (PtdSer) exposure on the plasma membrane, mediated by scramblase [1]. Necroptosis is caspase-independent cell death, regulated by RIPK3 and MLKL, and occurs when caspase activation is blocked. Pyroptosis depends on inflammatory caspases (1, 4, 5, 11), which cleave gasdermin D to form pores and release cytokines. Necroptosis, pyroptosis, and ferroptosis are programmed necroses, genetically regulated, but morphologically similar to necrosis. Necrosis involves rapid rupture of membranes and release of cellular contents, inducing strong inflammation. Ferroptosis, in turn, is driven by iron-dependent lipid peroxidation and loss of redox homeostasis [1]. Each type of cell death can play a crucial role in maintaining homeostasis and preventing disease.

In this review, we will focus on how apoptosis can impact homeostasis, host defense, and the activation of immune cells through metabolic reprogramming. These different types of cell death play fundamental roles in maintaining tissue homeostasis and in pathological conditions. Cell death by apoptosis can alter the tissue microenvironment by modulating the activation and function of phagocytes, as well as CD4 T lymphocytes in tolerogenic and infectious processes. In this review, we will discuss how the phagocytosis of apoptotic cells, called efferocytosis, contributes to maintaining the organism’s homeostasis, host defense, and metabolic reprogramming during.

## Efferocytosis of apoptotic cells—from recognition to degradation

**Fig. 1:**
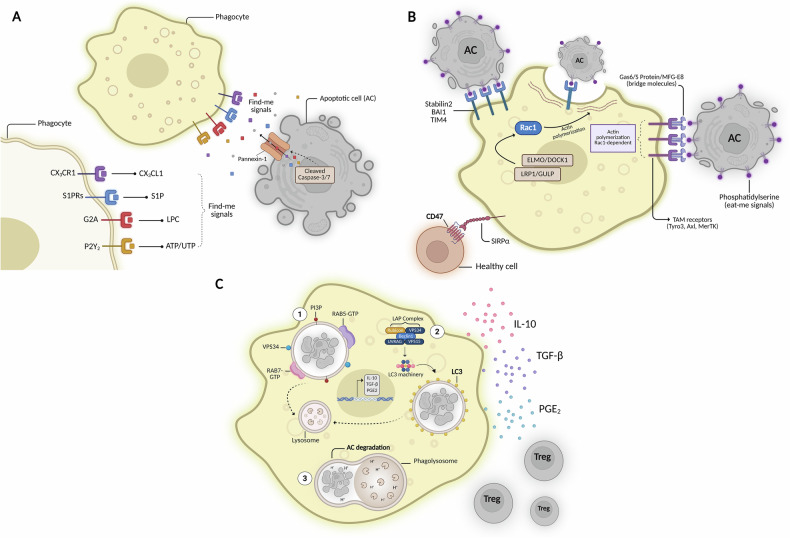
Stages of the efferocytic process. **A** Apoptotic cells release “find-me” signals to attract phagocytes. **B** “Eat-me” signals, such as PtdSer, are externalized on the plasma membrane and recognized directly or indirectly by efferocytic receptors expressed on phagocytes. The interaction of ACs through those receptors leads to a signaling pathway that culminates in actin filament polymerization, mediating AC internalization. **C** Phagosome maturation and subsequent fusion with the lysosome promote the degradation of the apoptotic body. The efferocytosis of non-infected ACs leads to the production of anti-inflammatory mediators and the differentiation of regulatory T cells. *Created with* BioRender.com.

The clearance of apoptotic cells (ACs) occurs through a process called efferocytosis, performed by professional phagocytes, including macrophages and dendritic cells (DCs), as well as non-professional phagocytes. This phenomenon characterizes an essential process for maintaining tissue homeostasis in physiological scenarios and restoring homeostasis after disease [1]. It has been estimated that the total number of cells in an adult body is approximately 37.2 trillion [2], and about 148 billion cells die daily. However, ACs are rarely found in different tissues, suggesting a rapid and efficient mechanism for removing those cells [3]. Efferocytosis is a highly orchestrated process in different stages, classified as (i) find-me, (ii) bind-me, (iii) eat-me, and (iv) digest-me signals. During the initial stages of the apoptosis process, the opening of pannexin-1 channels is activated by caspases-3/7. Through this mechanism, ACs begin to secrete soluble biochemical mediators into the extracellular environment, such as CX3CL1 (C-X3-C motif chemokine ligand 1), lysophosphatidylcholine, and ATP (adenosine triphosphate), called “find me” signals, which generate a chemoattractant gradient, favoring the encounter of phagocytes towards Acs [4]. After the approach of these phagocytes, recognition of the apoptotic body occurs through the interaction of molecules called “eat-me” signals, with the externalization of phosphatidylserine (PtdSer) into the external portion of the lipid bilayer in ACs, representing one of the essential signals during the recognition process [5]. Phospholipids are distributed asymmetrically in the plasma membrane, with PtdSer confined to the cytoplasmic portion of the plasma membrane. The distribution of phospholipids is regulated by flippase that is specifically responsible for the translocation of PtdSer from the outer to the inner portion of the lipid bilayer in an ATP-dependent manner. However, the induction of the apoptosis process leads to the activation of cleaved caspase-3, which in turn activates scramblases, which inhibit the activity of flippases. Flippase inhibition results in the irreversible exposure of PtdSer in the outer portion of the plasma membrane, making these cells permissive to phagocyte recognition [6]. In addition to distinguishing viable cells from ACs by exposure to PtdSer, ACs also begin downregulating the expression of “don’t eat-me” signals, such as CD47 and CD24 [7]. PtdSer recognition can occur through direct interaction with multiple efferocytic receptors expressed on phagocytes, such as TIM-1/4 [8], BAI-I [9], and Stabilin-2 [10]. Alternatively, PtdSer may interact indirectly with bridge molecules that mediate its interaction with efferocytic receptors, such as the glycoprotein MFG-E8, which links PtdSer to integrins αvβ5 and αvβ3, and the opsonins Gas6 and Protein S, which mediate its interaction with TAM family receptors (Tyro3, Axl, and MerTK) [11]. Signaling mediated by these receptors leads to the activation of Rac1, a GTPase of the RHO family, which regulates the polymerization and reorganization of the actin cytoskeleton in phagocytes, facilitating the formation of the phagocytic cup, the internalization of the apoptotic body and the formation of the phagosome - in a process mediated by the protein complexes LRP1-GULP and ELMO-DOCK, responsible for the activation of Rac1 [12, 13]. After recognition and internalization of the ACs, the phagosome containing the apoptotic “cargo” undergoes a maturation process mediated by the activity of VPS34 [14], phosphatidylinositol-3-phosphate, and Rab proteins, followed by the fusion of the late phagosome with the lysosome. Inside the phagolysosome, the apoptotic body is degraded by the action of proteases, phosphatases, and lipases - pH-sensitive catabolic enzymes [15]. In an alternative non-canonical pathway, specific components of the autophagy machinery - more specifically microtubule-associated protein 1 A/1B-light chain 3 (LC3) - are conjugated to lipids in the phagosome membrane, aiding in the formation of the phagolysosome, as well as in the efficient degradation of the apoptotic body [16]. The LC3-associated phagocytosis (LAP), a non-canonical pathway, lies at the intersection of autophagy and phagocytosis, promoting the release of ROS through NADPH oxidase. This process also involves Rubicon (RUN domain protein as Beclin-1 interacting and cysteine-rich containing) activity to stabilize the Class III PI3K complex, at the phagosome. The phagocytosis of microorganism, death cells, PtdSer binding or FcR promotes the recruitment of the Rubicon-containing PI3K complex (Beclin 1, VPS34, UVRAG) to the phagosome, where it ensures localized PI3P synthesis. Additionally, Rubicon stabilizes the NOX2 complex through interaction with p22phox, thereby sustaining ROS generation in phagocytic cells. LC3 conjugation onto the phagosome membrane enables its fusion with the lysosome, promoting the degradation of the phagosome’s content [17], demonstrating the versatility of efferocytosis, as it can utilize components of other cellular processes to clear apoptotic cells efficiently. Moreover, LAP regulates the polarization of tumor-associated macrophages (TAMs) into M2 cells (anti-inflammatory macrophages) and promotes the growth of Lewis lung carcinoma (LLC). The LAP-deficient macrophages recover the Type I IFN signaling and STING activation after phagocytosis of dying cells [18], suggesting LAP’s role in maintaining tolerance through apoptotic corpse degradation and impairing tumor regression and immunity. An overview of the efferocytic process steps is presented in Fig. 1.

## Essential role of efferocytosis in tissue homeostasis

Cellular renewal ensures the continuous clearance of apoptotic cells, preventing their accumulation and associated tissue damage. A classic example is the removal of aged neutrophils [19] and erythrocytes [20] by macrophages which occurs constantly in the body. Specialized phagocytes also contributed to maintaining the physiological function of different tissues, such as retinal pigment epithelial cells (RPE) and Sertoli cells (SCs). Previous studies demonstrate that MertK receptor and GAS6 and protein S (bridge molecules) are fundamental in the removal of opsin-containing outer segments (OS) of photoreceptor outer segments (POS) by retinal pigment epithelial (RPE) cells of the adult eyes. Animals deficient in MertK or Gas6 and protein S exhibit POS degeneration caused by impaired removal of these apoptotic cells [21]. Additionally, testosterone and other hormones are essential for maintaining spermatogenic homeostasis. However, the excess or deficiency of this hormone results in the death of germ cells. Sertoli cells are specialized phagocytes responsible for removing dead cells and debris. A deficiency in the removal of these germ cells in the seminiferous tubules can result in infertility in males. Male infertility is related to an impairment in the clearance of apoptotic cells [22]. During pregnancy, placental development involves intense apoptosis, and decidual macrophages and endothelial cells are the primary cells responsible for removing the apoptotic trophoblasts. The efferocytosis of those cells leads to a decrease of MHC-II as well as CD80, CD86, and CD40, and an increase of programmed death-1 ligand 1 (PD-L1) expression. In addition, the clearance of apoptotic trophoblasts causes the IL-10 production, and suppression of inflammatory cytokines by macrophages, suggesting that efferocytosis plays an important role in maternal-tolerance [23], while in the post-lactation apoptotic cell clearance at the mammary gland drives tissue remodeling [24]. In the central nervous system (CNS), during embryogenesis and adulthood, efferocytosis by ramified microglia balances cell death and neurogenesis, ultimately supporting brain development, optimizing neuronal connectivity, and ensuring that inflammatory responses remain appropriate and effective [25, 26]. As resident cells in intestinal crypts, Paneth cells (PCs) are essential for host defense by phagocytosing microorganisms, secreting antimicrobial peptides, and regulating the growth of enteric bacteria. Additionally, a recent study demonstrates that the deletion of PCs in vivo, using diphtheria toxin (DT) treatment, followed by the death of epithelial cells through irradiation, resulted in a profound decrease in the clearance of apoptotic cells. The new role of PCs as a key phagocyte during the clearance of ACs in the small intestine suggests that this phagocyte is responsible for preserving intestinal homeostasis and preventing inflammatory diseases [27]. Efferocytosis also regulates osteoclastogenesis and chondrogenesis, and its modulation may aid in disease management [28]. In healthy lungs, airway macrophages rapidly remove apoptotic cells, preventing the release of damage-associated molecular patterns (DAMPs), as well as self-antigens and alarmins, which can cause tissue damage and lead to diseases such as chronic obstructive pulmonary disease (COPD) [29], asthma [30], and COVID-19 [31]. Collectively, these findings highlight that efferocytosis is indispensable across multiple organ systems, supporting tissue integrity, immune balance, and overall physiological health.

## Efferocytosis outcomes on immune cells

### Efferocytosis of apoptotic cells suppresses the function of phagocytes

During efferocytosis of apoptotic cells, phagocytes develop a tolerogenic/resolution phenotype, characterized by the secretion of anti-inflammatory mediators [32]. In response to the efferocytosis of ACs, we and other research groups have previously demonstrated that macrophages [33, 34] and DCs [35, 36] predominantly produce TGF-β, IL-10, and prostaglandin E2 (PGE_2_). Recent findings emphasize the dying cell-macrophage interface as a modulator of macrophage functional heterogeneity. Understanding the specifics of this interaction, including the nature of apoptotic cells, the soluble factors produced, the density of signals (e.g., Ptsers), and the predominance of efferent receptors expressed by phagocytes in different tissues, appears to directly affect macrophage activation and polarization [37]. Liebold et al. (2024) [38] elegantly explored the impact of distinct dying cells on the functional diversity and gene expression of macrophages cultured in IL-4-enriched environments. Despite having similar levels of PtdSer exposed with varying degrees of caspase cleavage or absence of DNA break, the clearance of neutrophils, hepatocytes, and thymocytes elicited distinct outcomes such as tissue remodeling, tolerogenic effects, or minimal alteration of macrophage activation, respectively. Moreover, AXL and MERTK receptors are critically involved in the phagocytosis of apoptotic neutrophils and T cells, while hepatocytes require other efferocytic receptors. Additionally, apoptotic neutrophils and thymocytes exhibited similar lipidomic features, and their efferocytosis resulted in a higher accumulation of free fatty acids (FFAs) in macrophages, as well as the upregulation of genes associated with pathways activated by fatty acids and cholesterol, including PPARγ and LXR. These findings provide new insights into how efferocytosis affects effector function and phenotype, depending on the macrophage’s activation state, the source of the ACs, and the repertoire of efferocytic receptors. Furthermore, other studies have demonstrated the impact of efferocytosis on DCs maturation, significantly reducing the expression of costimulatory molecules and MHC-II [39]. The downregulation of molecules and the production of anti-inflammatory mediators are consequences of efferocytosis of ACs. As a result, some studies propose therapies based on the administration of ACs to suppress inflammatory responses in immune-mediated diseases, including diabetes [40], arthritis [41], colitis [42], and chronic obstructive pulmonary disease [43]. For example, in experimental arthritis, it has been demonstrated that the systemic administration of ACs protected mice from autoimmune arthritis by inducing regulatory B cells, thereby restraining inflammatory responses [41]. Similarly, apoptotic cell treatment improves colitis by suppressing NF-κB activation and inflammasome signaling, resulting in reduced production of pro-inflammatory cytokines and tissue damage [42]. Together, these studies provide evidence that apoptotic cell administration could be an alternative and promising approach for immunomodulating immune homeostasis in chronic inflammatory diseases.

### Efferocytosis during infectious diseases

Cell death is a common consequence of infection, and pathogens can benefit from the anti-inflammatory outcome. During the infection, the pathogen can infect tissue or immune cells, inducing the production of cytokines and chemokines, which allows the influx of inflammatory cells to the site of infection. After hours or days, those cells may progress through a process of cell death, leading to various cell death patterns, such as apoptosis, necrosis, or pyroptosis. This process can either suppress immunity or improve host defense during infections, depending on the nature of cell death [44].

### Suppression of the host defense

Regarding the immunosuppression caused by the accumulation of ACs, infection with cytopathic vesicular stomatitis virus (VSV) enhances the number of ACs in the spleen. The pretreatment of BMDCs with dead cells led to TGF-β and IL-10 production through MerTK, and a reduction in IFN-α levels, suggesting that dead cells induced by VSV infection induce innate anergy and limit the antiviral immune response [44]. The efferocytosis machinery can also serve as a mechanism for some pathogens to gain access to cellular compartments and infect new cells. During infection with *Listeria monocytogenes*, which secretes listeriolysin O, a pore-forming toxin, the bacteria can escape the phagosome and release cellular vesicles coated with PtdSer. In turn, these infected vesicles act as a “Trojan horse” when recognized and phagocytosed by macrophages, facilitating bacterial dissemination [45]. In the experimental model of Chagas’ disease, one injection of apoptotic cells (i.p.) after 7 days of infection with *Trypanosoma cruzi*, increases parasitemia in vivo. Moreover, co-cultures of peritoneal macrophages with apoptotic cells, followed by infection with *T. cruzi*, increase parasite replication. This suppressive effect is mediated by TGF-β and PGE_2_, as the treatment of infected mice with indomethacin drastically reduces the parasitemia [46]. The production of PGE_2_ during efferocytosis leads to the suppression of the phagocytosis and killing function of alveolar macrophages, in vitro, via EP2–adenylyl cyclase–cAMP pathway. Moreover, the instillation of ACs followed by *Streptococcus pneumoniae* infection increased the bacterial load and led to bacterial dissemination, in vivo, via EP2 prostanoid receptors [34].

### Improvement of antimicrobial defense

Efferocytosis can also help the host defense mechanism against various pathogens. During *Mycobacterium tuberculosis* infection, the clearance of bacteria depends on the phagocytosis of infected apoptotic macrophages and subsequent lysosomal degradation of the apoptotic corpse [47]. A similar mechanism was observed during the *Salmonella* Typhimurium infection, which leads to macrophage death by pyroptosis. These pyroptotic macrophages release debris containing the damaged bacteria that are engulfed by newly recruited neutrophils, resulting in pathogen clearance [48]. Efferocytosis of dead infected cells can also represent an essential source of antigens to activate T cells. During herpesvirus infection, CD8α dendritic cells recognize and internalize ACs via AXL, LRP-1, and RANBP9 mechanisms and cross-present them to CD8 T lymphocytes. The absence of those receptors impaired efficient OT-I lymphocyte activation and increased host susceptibility to death [49]. Phagocytosis of infected apoptotic cells can also favor the activation and recruitment of CD4+  T lymphocytes. The efferocytosis of apoptotic cells infected with *Escherichia coli*, in vitro, activates TLR4/TRIF and Myd88 and induces TGF-β, IL-6, and IL-23 production by dendritic cells, leading to the differentiation of IL-17A⁺ lymphocytes. In addition, *Citrobacter rodentium* infection induces intense apoptosis in epithelial cells and promotes Th17 differentiation to enhance bacterial clearance [50]. However, we demonstrate that, in addition to those cytokines, the efferocytosis of *E. coli*-infected apoptotic cells also triggers the abundant production of PGE_2_ and IL-1β by BMDC in vitro. The presence of PGE_2_ restrains Th17 differentiation through EP(4) prostanoid receptors. Moreover, treating mice with indomethacin, a non-selective cyclooxygenase inhibitor or EP4 antagonist, increased Th17 cell differentiation and enhanced the production of antimicrobial peptides, thereby reducing the bacterial load in the gut and improving host defense against *C. rodentium* infection [36]. Nonetheless, during methicillin-resistant *Staphylococcus aureus* (MRSA) skin infection, rescuing PGE_2_ effects in diabetic mice, using a topical treatment with misoprostol, rescued DC migration to draining lymph nodes, Th17 differentiation, and improved host defense [51] suggesting that PGE_2_ effects during efferocytosis of infected cells are context-dependent. During infection, the clearance of microorganisms, as well as dead cells, helps prevent the development of autoimmunity. It has been demonstrated that DCs can favor the presentation of non-self antigens over self-antigens. The phagosome containing the microorganism, or a Toll ligand, signals through the proteolysis of the p31 invariant chain and becomes capable of forming complexes with MHC-II molecules. In contrast, ACs-containing phagosomes lack this ability [52]. However, when DCs engulfs the apoptotic cell containing the microorganism, the cell loses the ability to distinguish between self- and non-self-antigens, since both components are directed to the same phagosome. Our group demonstrated that efferocytosis of apoptotic cells infected with *E. coli* by BMDCs leads to an increase in MHC-II, CD86, and CCR7, as well as the production of IL-6, IL-23, and IL-1β [35]. An elegant study demonstrated that apoptotic cells generated by *C. rodentium* infection can inhibit bacterial load. However, the presentation of autoantigens can also promote both the generation of autoreactive Th17 cells and an IgA response against these self-antigens, resulting in tissue damage in the intestine [53]. This demonstrates that the efferocytosis of infected cells is beneficial for an effective assembly of the adaptive immune response but can also contribute to the development of autoimmune disease.

Different microorganisms activate distinct sets of pattern recognition receptors, leading to the production of specific inflammatory mediators by DCs. Our group has previously reported that the type of pathogen present within ACs can induce the production of different soluble mediators by BMDCs during efferocytosis. While the recognition of *E. coli* within the apoptotic body led to Th17 cell differentiation, the supernatant from the efferocytosis of apoptotic cells infected with *Streptococcus pneumoniae* favored the activation of Th1 lymphocytes. The recognition of pathogen-associated molecular patterns by the adaptor molecule RIP2 led to high levels of IL-1β, which was crucial for stimulating the production of IFN-γ by Th1 cells [54].

The presence of macrophages in several tissues plays a crucial role in resolving infections and removing dead cells resulting from the infectious process. The efferocytic process of infected or non-infected ACs can drive polarization of macrophages and potentially lead to either exacerbation or suppression of the inflammatory process. A recent study has shown that SARS-CoV-2 infection induces intense apoptosis, and the efferocytosis of SARS-CoV-2-infected ACs promotes the production of IL-6 and IL-1β, thereby exacerbating the “cytokine storm”. Previous studies have shown that efferocytosis and degradation of ACs corpse improve the capacity of continual efferocytosis by macrophages. However, the efferocytosis of SARS-CoV-2-infected ACs by macrophages inhibits many efferocytic receptors, such as CD36, αVβ5 integrin (ITGB5), and T cell immunoglobulin mucin receptor 4 (TIM4), as well as MER proto-oncogene tyrosine kinase (MERTK). Moreover, those macrophages lose the ability to maintain the continual efferocytosis, promoting the accumulation of ACs, causing tissue damage associated with a dysfunctional efferocytosis [31].

Efferocytosis of apoptotic cells infected with *S. pneumoniae* or *Klebsiella pneumoniae* (*S. pneumoniae*-AC and *K. pneumoniae*-AC, respectivelly) generates distinct macrophage polarization profiles in vitro and in vivo. *S. pneumoniae*-AC induces a mixed phenotype, increasing *Ccr7, IL-1β*, *Fizz1* and NO, versus *K. pneumoniae*-AC which promotes a predominantly inflammatory profile, with reduced *Arg1, Cd206* and elevated nitrite, TNF-α, IL-1β. In vivo, F4/80⁺ and alveolar macrophages from mice receiving *K. pneumoniae*-AC show higher *Cd86, Nos2*, lower *Cd206*, and lung homogenates display increased IL-1β, IL-6, TNF-α compared to PBS. In an OVA asthma model, *S. pneumoniae*-AC decreases *Arg1, Cd206, Ym1*, increases *Fizz1, Nos2* in alveolar macrophages, and reduces IL-1β, IL-12 in BALF relative to PBS-OVA-AC. Together, these studies highlight that the outcome of efferocytosis of infected apoptotic cells is strongly influenced by the nature of the pathogen cargo as well as the identity of the phagocyte, underscoring the context-dependent plasticity of efferocytic responses [55].

Efferocytosis of infected dying cells involves handling cell micro- and macromolecules, including carbohydrates, amino acids, and nucleic acids. Similarly, it has been demonstrated that the phagolysosomal degradation of phagocytosed bacteria can provide carbon atoms and amino acids for the biosynthesis of itaconate and glutathione in macrophages. An elegant study demonstrates phagolysosome degradation of killed bacteria, independent of the bacterial species used. It provides an alternative nutrient source of metabolites, such as amino acids and other essential nutrients, that support mitochondrial function and protein synthesis in macrophages more effectively than live bacteria. In conclusion, the degradation of killed bacteria contributes more efficiently to improving cell viability under nutrient-deprived conditions than live bacteria, stimulating pathways such as glutathione (GSH) biosynthesis, compared to live bacteria [56].

**Fig. 2:**
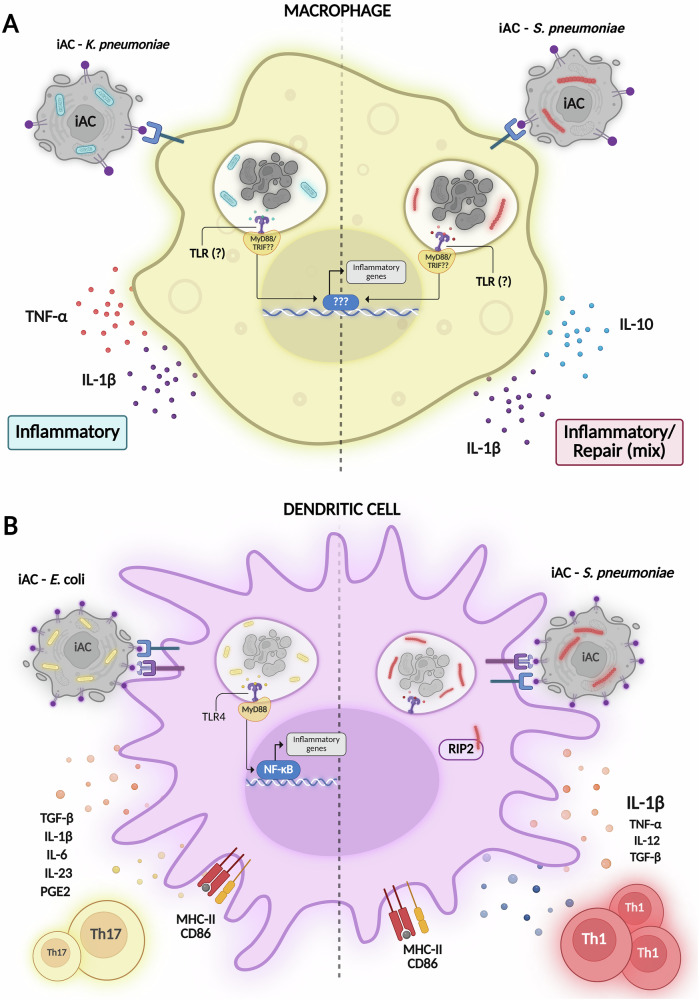
Efferocytosis during infectious diseases. **A** Macrophage and **B** dendritic cell activation profiles during the efferocytosis of infected apoptotic cells (iAC) with *Klebsiella pneumoniae*, *Streptococcus pneumoniae*, *and Escherichia coli*, as previously described. *Created with* BioRender.com.

Despite advances in the understanding of the modulation of metabolism during phagocytosis of eukaryotic and prokaryotic cells, the impact of recognition of PAMPS and self-components altogether during efferocytosis of infected cells on the modulation of metabolic pathways in the efferocyte remains elusive. Figure 2 shows an overview of the immune outcomes of efferocytosis during infections.

### Overview of the major metabolic pathways

The products generated from the activation of the different metabolic pathways are shared and used in interdependent anabolic and catabolic pathways, allowing the integrated functioning of these pathways to support the phenotype and the activation of immune cells. In the field of immunometabolism, the main pathways studied in different contexts of immune responses include glycolysis, the pentose phosphate pathway (PPP), the tricarboxylic acid cycle (Krebs cycle), fatty acid oxidation (β-oxidation), and fatty acid synthesis (FAS) and amino acids metabolism.

#### Glycolysis

The glycolytic pathway takes glucose via GLUT transporters and phosphorylates it to glucose-6-phosphate (G6P) by hexokinase (HK), consuming ATP. This metabolism offers benefits such as (i) reduction of NAD+ to NADH, essential for several enzymes, and (ii) supply of nucleotide intermediates (PPP), serine biosynthesis, and de novo lipogenesis. The glycolytic flux is maintained by converting pyruvate to lactate, regenerating NAD + , and exporting lactate via MCTs. In aerobic glycolysis, pyruvate generates acetyl-CoA for the Krebs cycle. Despite the low energy yield (2 ATP per glucose), glycolysis is fast and activated in proliferative or stressed cells [57].

#### Pentose phosphate pathway (PPP)

The PPP occurs in the cytosol, parallel to glycolysis, diverting G6P to produce precursors essential for cell growth. This pathway is divided into an oxidative phase, which generates NADPH for lipid synthesis, antioxidant defense, in addition to ribulose-5-phosphate and intermediates for the synthesis of amino acids, and in phagocytes, NADPH also supplies NADPH oxidase, indirectly contributing to the production of ROS. The non-oxidative phase, which converts ribulose-5-phosphate into ribose-5-phosphate for nucleotides and nucleic acids, as well as glycolytic intermediates such as fructose-6-phosphate and G3P. G3P can be converted to dihydroxyacetone via mitochondrial GPD2, transferring electrons to mitochondrial complex III, reintegrated into glycolysis, or used in gluconeogenesis [58].

#### Tricarboxylic acid cycle (TCA)

The TCA (Krebs) cycle is central to energy metabolism, integrating metabolic pathways via cataplerotic (diverting intermediates for biosynthesis) and anaplerotic (replacing intermediates) reactions. In the mitochondria, acetyl-CoA (derived from pyruvate or fatty acids) combines with oxaloacetate to form citrate, which can be exported or oxidized in the cycle. Glutamate also provides carbon to the TCA via conversion to α-ketoglutarate. Oxidation in the cycle generates NADH and FADH2, which are used in the electron transport chain to drive OXPHOS. The generated proton gradient moves H+ through ATP synthase, converting ADP to ATP. Although slower than glycolysis, this pathway is highly efficient, generating 30-32 ATP per glucose [59].

#### Fatty acid oxidation (FAO)

FAO is a crucial catabolic and anaplerotic pathway by generating products for feeding the TCA cycle, such as acetyl-CoA, NADH, and FADH2. The first step of FAO occurs in the cytosol and consists of activating fatty acids by forming an acyl-CoA. Short-chain acyl-CoA ( ~ 5 carbons in the aliphatic tail) diffuses passively across the mitochondrial membrane. On the other hand, long-chain acyl-CoA (C12–C20) requires a carnitine shuttle system to transfer these molecules into the mitochondria. In the mitochondrial matrix, β-oxidation cycles begin, which reduce the length of the fatty acid into two-carbon fragments per cycle, releasing acetyl-CoA, NADH, and FADH2. This process continues until the fatty acid is completely converted into multiple molecules of acetyl-CoA. Acetyl-CoA then enters the Krebs cycle, where it is oxidized. At the same time, the reducing equivalents (NADH and FADH2) feed the electron transport chain during oxidative phosphorylation (OXPHOS) to generate most of the cellular ATP [60].

#### De novo fatty acid synthesis (FAS)

The FAS pathway is a key player in meeting the metabolic demands during lipid biosynthesis, integrating glycolysis, TCA, and PPP intermediates. Citrate exported from TCA to the cytosol is transformed to acetyl-CoA and oxaloacetate by ATP-citrate lyase. The acetyl-CoA carboxylase catalyzes the carboxylation of acetyl-CoA to malonyl-CoA, a key precursor of FAS. Fatty acid synthase, an enzyme that acts in an NADPH-dependent manner, catalyzes the synthesis of fatty acids, such as palmitate, a key product in the composition of membrane phospholipids, in protein acylation, and as a precursor in cholesterol synthesis [58].

## Metabolic reprogramming of phagocytes during efferocytosis of apoptotic cells

In the last few years, the concept of efferotabolism has been proposed to describe the metabolic reprogramming that occurs within phagocytes following the engulfment of apoptotic cells [61]. The interest of the new area lies in investigating the correlation of metabolism and immunological aspects of efferocytosis, involved during the degradation of apoptotic corpses, which requires an adaptation of lipid, carbohydrate, and amino acid metabolism acquired during the degradation of ACs. Most of the current knowledge on efferotabolism has been derived from studies in atherosclerosis, where defective apoptotic cell clearance contributes to chronic inflammation and plaque progression. However, the principles established in this context, such as the need for metabolism to sustain continuous efferocytosis and the coupling of catabolic and anabolic pathways to support anti-inflammatory signaling, are important for physiological and pathological conditions during apoptotic cell clearance [62]. Since the introduction of this new concept, several studies have investigated how specific metabolic pathways are engaged during efferocytosis to sustain successive rounds of apoptotic cell uptake and degradation. Efferocytosis of ACs by phagocytes, such as macrophages and DCs, requires dynamic orchestration of several metabolic pathways during the different stages of efferocytosis [63]. The ability of a phagocyte to perform multiple “rounds” of phagocytosis of ACs in a relatively short time—called continuous efferocytosis—is essential in scenarios where the ACs:phagocytes ratio is high, such as after an acute inflammatory process or tissue injury. To do so, different steps are necessary, namely: (i) recycling of the plasma membrane and efferocytic receptors due to the formation of the phagosome, allowing new “rounds” of efferocytosis and (ii) processing the macromolecules, such as lipids, carbohydrates, amino acids and nucleic acids, resulting from the degradation of ingested ACs which need to be rapidly metabolized or eliminated [64].

### Fatty acid metabolism and OXPHOS

**Fig. 3:**
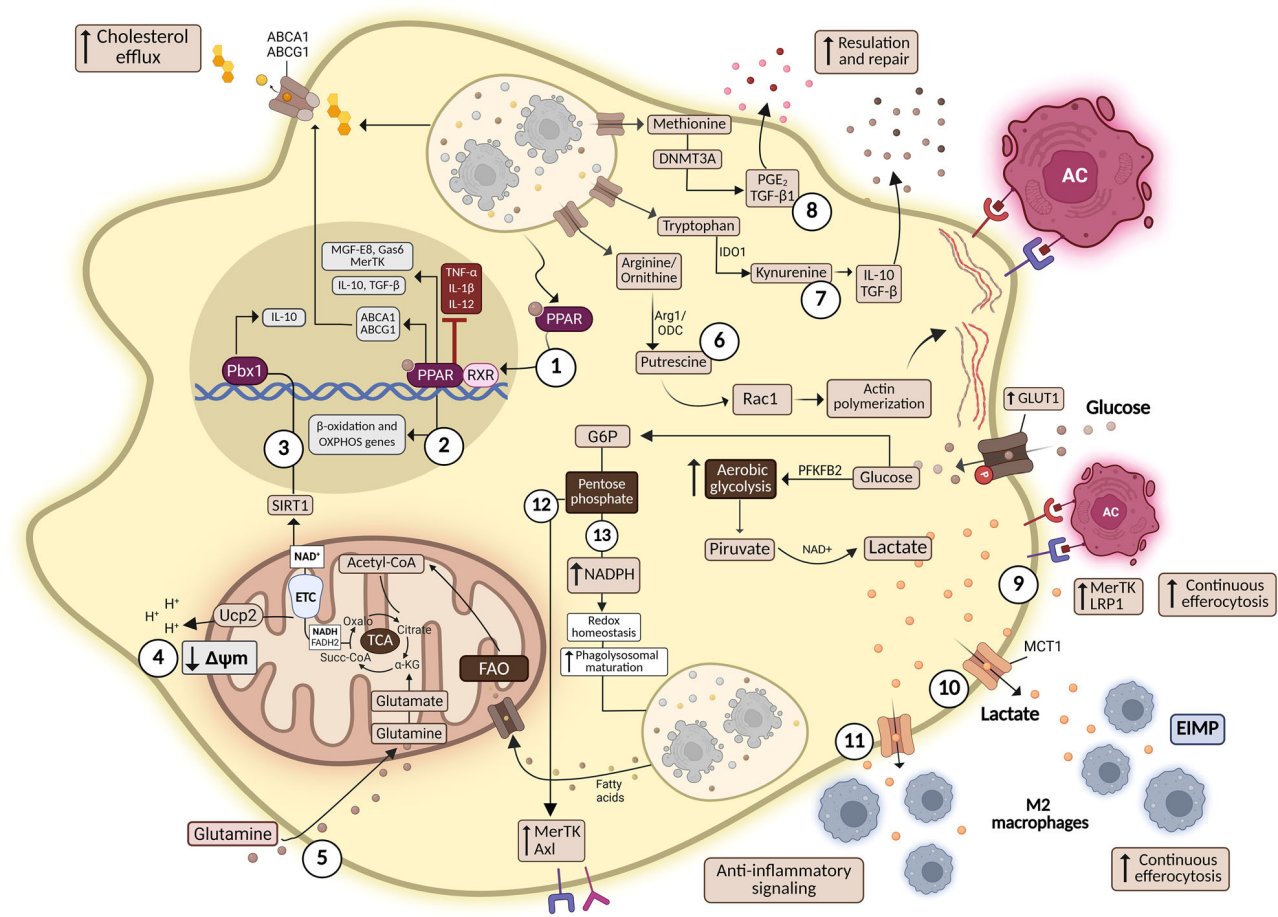
Metabolic reprogramming in macrophages during the efferocytosis of non-infected ACs. The process of efferocytosis and the release of metabolites derived from the degradation of apoptotic cells modulate metabolic signaling in macrophages to mediate inflammation resolution, sustain continuous efferocytosis, and protect phagocytes from the excess of macromolecules originated from the apoptotic cargo. *Created with* BioRender.com.

Previous studies have shown that sterols derived from ACs activate transcription factors and nuclear receptors, including PPARγ and Liver X receptor alpha (LXRα). This activation is responsible for regulating lipid homeostasis by promoting the increased expression of the membrane transporter channels Abca1 and Abcg1, which control cholesterol efflux and thereby prevent lipid toxicity and cellular dysfunction [65]. In addition to protecting macrophages from free cholesterol overload, activation of PPAR and LXRα promotes the transcriptional regulation of efferocytic receptors, such as MerTK, and opsonins, including MFG-E8 and Gas6 [66]. Furthermore, the interaction of ACs with these nuclear receptors promotes the synthesis of IL-10 and TGF-β, while downregulating the production of TNF-α, IL-1β, and IL-12 in macrophages [66–68] (Fig. 3–Item 1). Mitochondria are the central organelle capable of using the lipid load derived from the degradation of ACs for energy generation. In response to lipid overload, PPARγ activation stimulates mitochondrial biogenesis through PGC-1α, thereby increasing the mass and functionality of mitochondria. In addition, PPARγ activation promotes the expression of genes related to β-oxidation and oxidative phosphorylation, allowing phagocytes to efficiently utilize lipids derived from efferocytosis as an energy source [69, 70] (Fig. 3–Item 2). These findings support the crucial role of mitochondrial metabolism in modulating efferocytosis and preventing inflammation. In macrophages isolated from mouse myocardium after infarction and in bone marrow-derived macrophages (BMDMs) exposed to ACs in vitro, the concentration of intracellular fatty acids and mitochondrial oxygen consumption upregulated β-oxidation and OXPHOS, leading to the generation of NAD+ and promoting the activation of SIRT1 (a sirtuin) that targets PPARγ activity and genes related to mitochondrial biogenesis [71, 72]. This activation leads to the binding of PBX1 to the Il10 promoter, resulting in increased production of IL-10, which contributes to tissue repair [73] (Fig. 3–Item 3). Moreover, after the efferocytosis of ACs by macrophages during atherosclerosis, macrophages showed increased β-oxidation and mitochondrial respiration in a lipin-1-dependent manner, a phosphatase with a transcriptional co-regulatory activity related to the expression of genes involved in fatty acid transport and lipid metabolism [74, 75]. Fatty acid oxidation favored the polarization of macrophages towards a resolution/repair profile in response to efferocytosis of ACs or IL-4 stimuli, with lipin-1 activity being critical for establishing a reparative and pro-resolution phenotype in macrophages in the atherosclerotic plaques [76]. Lipin-1 deficiency in macrophages results in reduced β-oxidation activity and oxygen consumption rates, which leads to impaired continuous efferocytosis, highlighting the importance of oxidative metabolism in supporting cellular functions during the clearance of dead cells [74]. Based on these findings, it seems that β-oxidation has the potential to provide energy more efficiently than other mechanisms, enabling continuous efferocytosis. OXPHOS activation may not be solely associated with ATP production in efferocytic macrophages. Studies have shown that mitochondrial uncoupling protein 2 (Ucp2) is upregulated in phagocytes that internalize ACs [77]. Increased Ucp2 favored proton leakage across the inner mitochondrial membrane, depolarizing mitochondria and uncoupling OXPHOS from ATP synthesis, reducing the mitochondrial membrane potential.

Macrophages from Ucp2-deficient mice exhibited impaired efferocytosis in vitro and in vivo, suggesting that an excessive increase in mitochondrial membrane potential impairs the ability of macrophages to maintain continual efferocytosis of ACs. However, the mechanism that regulates this process remains unknown [78] (Fig. 3–Item 4). Interestingly, mitochondrial fission, mediated by the GTPase Drp1, has been shown to promote the continued clearance of ACs by macrophages. This mechanism enables macrophages to sustain focal exocytosis and efficiently internalize multiple ACs in sequence, providing a mechanistic explanation for how mitochondrial dynamics support continuous efferocytosis [79]. Thus, while β-oxidation and OXPHOS are central to supporting efferocytic activity in macrophages, recent evidence extends the role of lipid metabolism to dendritic cells. In conventional DCs (cDCs), cholesterol acquired via both de novo synthesis and efferocytosis is mobilized to form plasma membrane lipid nanodomains. These domains enhance DCs maturation in response to stimuli, such as TLR agonists, and stabilize MHC class II and IFN-γR expression, thereby ensuring efficient signaling and immunogenic function. Cholesterol transport critically depends on NPC1, whereas the tyrosine kinase receptor AXL negatively regulates the process: its deletion enhances nanodomain formation, promotes cDC maturation, and increases antitumor immunity [80]. These findings bring new knowledge about how efferocytosis is more than a process that promotes the clearance of ACs, thereby impairing tissue damage. This process also supplies cholesterol, which is essential for cDC maturation, as a central metabolic regulator of immune responses.

### Glutaminolysis

In addition to pyruvate from glycolysis and acetyl-CoA generated from fatty acid oxidation, other conventional or alternative pathways can be utilized to supply the TCA cycle and, consequently, maintain cellular function. Recently, it has been demonstrated that glutaminolysis, which also supplies substrates for the Krebs cycle, facilitates the activation of OXPHOS and ATP production, thereby regulating efferocytosis efficiently through a non-canonical glutamine metabolism. In response to ACs, glutaminase-1 is upregulated, which promotes glutamate production and increases α-ketoglutarate. In this study, the absence of glutaminase-1 in macrophages impaired efferocytosis both in vitro and in vivo, and exacerbated atherosclerosis, a condition in which the efficient clearance of apoptotic cellular debris is essential to limit disease progression [81] (Fig. 3–Item 5). Another study supports the idea of interplay between apoptotic cell identity, metabolic status, and phagocyte receptors in determining the degradation pathway and the immunological outcomes of efferocytosis. Better et al. 2025 demonstrates that the type of apoptotic cell engulfed, neutrophils or epithelial cells, modulates the ability of alveolar macrophages to respond to bacterial infection. During inflammation, this phagocyte exhibits impaired antibacterial but improved pro-resolution functions. Efferocytosis of apoptotic neutrophils reprogrammed macrophage mitochondrial metabolism through mieloperoxidase-dependent UCP2, which increases proton conductance in the inner mitochondrial membrane, thereby impairing reactive oxygen species (mtROS) production in response to bacteria. In contrast, the epithelial cells’ efferocytosis confers antibacterial abilities. Thus, the identity of ACs, together with the upregulated expression of receptors such as UCP2, orchestrates immunometabolic states that limit phagocyte plasticity and prioritize inflammatory resolution and tissue protection over antibacterial defense [82].

### Amino acid metabolism

Like fatty acids, amino acids derived from the degradation of ACs also play essential roles in regulating phagocyte polarization and function. In this context, elegant studies published by Ira Tabas’s group have contributed to the understanding of cellular metabolism by demonstrating that macrophages use amino acids, such as arginine and ornithine, derived from the degradation of ACs to synthesize putrescine that activates Rac1, which stabilizes the machinery required for actin remodeling and plasma membrane projection, supporting continuous efferocytosis. Consistently, deficiency of arginase 1—which converts arginine to ornithine—or deficiency in ornithine decarboxylase—which catalyzes the conversion of ornithine to putrescine—in myeloid cells, is associated with efferocytic dysfunction and failure to resolve atherosclerosis in a murine model [64] (Fig. 3–Item 6). In addition to arginine, it was demonstrated that tryptophan converted to kynurenine, catalyzed by the enzyme indoleamine 2,3-dioxygenase 1, induced the expression of IL-10 and TGF-β in macrophages, thereby increasing continuous efferocytosis and favoring tolerance both in vitro and in vivo [83] (Fig. 3–Item 7). Additionally, the methionine derived from ACs is metabolized to S-adenosylmethionine, which supports DNMT3A-dependent DNA methylation, driving epigenetic reprogramming and inducing the production of pro-resolving mediators such as PGE₂ and TGF-β1, which lead to resolving inflammation in models such as sterile peritonitis, thymic injury, and atherosclerosis [84] (Fig. 3–Item 8). These findings provide us with an important new insight into how amino acids generated during efferocytosis serve not only as metabolic substrates, but also as modulators of macrophage function and preservers of tissue homeostasis.

### Glycolysis

A recent study has challenged the view that glycolysis is exclusively associated with the inflammatory metabolism of macrophages. However, the mechanisms that regulate glycolysis in inflammatory macrophages and those in efferocytosis processes are distinct. In macrophages stimulated with LPS and IFN-γ, inhibition of OXPHOS and increased glycolysis is a process that prevents differentiation towards a resolution/repair profile when stimulated with IL-4 [85]. In contrast, another demonstrated that macrophages, during the efferocytosis process, present a transient increase in glycolysis ( ~ 1h-6h), that is dependent on rapid post-translational mechanisms with increased expression of GLUT1 on the cell surface and glucose uptake, in a process that involves the activation of the glycolytic enzyme fructose-2,6-bisphosphatase 2 (PFKFB2). The authors also demonstrated that glycolysis-derived products regulate the expression of MerTK and LRP1, thereby sustaining the continuous efferocytosis of ACs [86] (Fig. 3–Item 9). Intriguingly, recent research has shown that lactate is not only essential for efferocytosis but also for sustaining efferocytosis-induced macrophage proliferation, a process responsible for increasing the number of macrophages capable of performing continuous efferocytosis, thus contributing to the resolution of inflammation and tissue repair [87] (Fig. 3–Item 10). Consistently, Morioka and collaborators observed that efferocytosis of ACs by macrophages increases glycolytic activity while reducing fatty acid oxidation. Their elegant study demonstrated that metabolites from ACs activate the enzyme SGK1 in macrophages, upregulating the expression of Glut1 and Mct1. The uptake of extracellular glucose via GLUT1 favors increased aerobic glycolysis, and lactate, generated as a byproduct of this oxidation, which is secreted via MCT1. The presence of this extracellular lactate promotes the polarization of macrophages towards a resolution/repair phenotype, which contributes to tissue remodeling and immune tolerance [88] (Fig. 3–Item 11).

### Pentose phosphate pathway (PPP)

In addition to glycolysis, the PPP is markedly upregulated in thymic resident macrophages during efferocytosis of ACs. As demonstrated, PPP activation not only regulates the expression of Axl and MerTK but also supplies NADPH to sustain redox homeostasis under efferocytic stress but also regulates the expression of apoptotic cell sensors and maintains membrane fluidity, features that are intrinsically linked to efficient efferocytosis [89] (Fig. 3–Item 12). However, the role of the PPP in efferocytosis is not straightforward and remains controversial. Although the oxidative phase of the PPP has been shown to support efferocytosis through NADPH generation, reports indicate that efferocytosis reduces the expression of PPP-related genes, thereby decreasing the flux of the PPP pathway [90]. Furthermore, treatment of macrophages with a PPP agonist in vitro has been shown to impair efferocytosis and exacerbate autoimmune disease in an experimental model of systemic lupus erythematosus [91]. Macrophages tend to accumulate in hypoxic regions, which can modulate the function of this phagocyte. Notably, chronic low-oxygen conditions have been shown to enhance macrophage efferocytosis. This effect is supported by increased glucose flux through the noncanonical PPP, resulting in higher NADPH production, which promotes lysosomal maturation and maintains intracellular redox balance, thereby facilitating more efficient clearance of ACs [92] (Fig. 3–Item 13). An overview of the metabolic pathways activated during efferocytosis is summarized in Fig. 3.

Like macrophages, DCs play a key role in the clearance of ACs, modulating the immune response in different contexts, whether under non-infected conditions or during the removal of infected ACs. An elegant study has shed light on an underappreciated role of DCs in promoting wound healing through efferocytosis and metabolic reprogramming. RNA sequencing of efferocytic BMDCs revealed the upregulation of the Slc7a11 gene, cystine/glutamate transporter, that regulates intracellular glutamate for extracellular cysteine exchange and is highly expressed in skin-resident DCs. Slc7a11 depletion selectively boosted BMDCs’ efferocytosis (in vitro) or type 1 conventional DCs (ex vivo) by allowing DCs to use intracellular glycogen levels to supply glycolytic pathway. In a full-thickness skin wound model in db/db mice, pharmacological inhibition of SLC7A11 rescued DCs’ efferocytosis and significantly improved wound closure [93].

Despite the enormous contribution in recent years to understanding cellular metabolism associated with efferocytosis, especially regarding its impact on the effector functions of macrophages and, more recently, dendritic cells in physiological, inflammatory, and pathological contexts, there are still important gaps that remain unknown. In particular, it remains poorly understood how different metabolic pathways modulate the phagocytic and microbicidal potential of phagocytes during the clearance of infected ACs in different infection models.

## Concluding remarks

Efferocytosis is essential for maintaining the vital functions of different organs and preventing tissue damage resulting from the accumulation of ACs, which are efficiently removed daily by macrophages, dendritic cells, and non-professional phagocytes.Efferocytosis is also crucial for the polarization of macrophages, the activation of dendritic cells, and the driving of T-celldifferentiation during homeostasis and host defense. Different bacterial cargos inside of ACs can either enhance pathogen clearance or inadvertently facilitate immune evasion. Moreover, efferocytosis of infected ACs may expose self-antigens, potentially contributing to tolerance breakdown and triggering autoimmune diseases. The activation of different metabolic pathways during the various stages of the efferocytic process, as well as the products generated during the degradation of apoptotic cells, necessitates an adaptation of lipid, carbohydrate, and amino acid metabolism, enabling the integrated functioning of these pathways to sustain the phenotype and activate immune cells. Therefore, further studies to clarify the metabolic pathways involved in initiating and maintaining the tolerogenic or immunogenic states of dendritic cells and macrophages are relevant to understanding adaptive immune outcomes in disease resolution or progression related to efferocytosis defects. Thus, exploring the metabolic mechanisms involved becomes intriguing in the search for targeted therapies to enhance the immune response and protect against immune-mediated diseases. However, the specific gaps remain unanswered regarding which metabolic pathways are involved during the efferocytosis of infected ACs, which could maintain the immunogenic phenotype to clearance the infection, and these remain poorly explored. Moreover, whether professional and non-professional efferocytes rely on distinct metabolic programs during efferocytosis, and how different forms of cell death alter phagocyte metabolism. This knowledge may provide a better understanding of how cell metabolism in the efferocytosis process can integrate tolerance, inflammation, or antimicrobial immunity.
